# Analysis of the Coverage of Paratriathlon and Paratriathletes in Canadian Newspapers

**DOI:** 10.3390/sports6030087

**Published:** 2018-08-29

**Authors:** Gregor Wolbring, Brian Martin

**Affiliations:** Department of Community Health Sciences, Cumming School of Medicine, University of Calgary, 3330 Hospital Drive NW, Calgary, AB T2N 4N1, Canada; brian.martin1@ucalgary.ca

**Keywords:** paratriathlon, paratriathlete, parasport, para-athlete, sport, Olympics, Paralympics, social responsibility, legacy goals, Canadian newspapers

## Abstract

From recreational to elite levels, sport has many benefits for disabled people. At the same time, it is acknowledged that there is a trickle-down problem from para-elite sport to sport participation of disabled people, in general. Newspapers are one form of media that sets agendas and influences public opinion. Many studies have highlighted problematic aspects of parasport and para-athlete coverage in newspapers. Paratriathlon was one of two new events added to the Paralympics in Rio 2016, which increased its visibility in the public domain. We investigated the coverage of paratriathlon and paratriathletes in 300 Canadian newspapers using the ProQuest database Canadian Newsstream as a source, and utilizing a descriptive quantitative and a qualitative thematic content analysis. The main themes evident in the reporting on paratriathlon and paratriathletes, in the three hundred Canadian newspapers we covered, were the supercrip imagery of the para-athlete, personal stories mostly linked to the supercrip imagery, and the theme of able-bodied athletes in juxtaposition to the para-athletes. Using the lens of the four legacy goals of the International Paralympic Committee, we conclude that our findings are detrimental to the fulfillment of the four legacy goals.

## 1. Introduction

Sport, from the recreational to the elite level, has an effect on many humans, whether in their role as spectators or participants [1,2,3], including disabled people [1,4,5]. It is, for example, noted that sport can contribute to the personal and moral development of individuals participating in sport, if done right [6]. Article 30 of the Convention on the Rights of Persons with Disabilities highlights the importance of all forms of sport for disabled people [7]. At the same time, it is acknowledged that there are attitudinal and structural barriers to the inclusion of disabled people in sport, in general [8]; that there is little trickle-down benefits from para-elite sport, to sport and exercise performed by disabled people on the non-elite level [9,10,11] and numerous problems of how Parasport and para-athletes are portrayed, for example, in the media, are recognized [12,13]. The International Paralympic Committee generated four components of a legacy they expect from hosting a given Paralympic event: (1) accessible infrastructure in sport facilities and in the overall urban development; (2) development of sport structures/organizations for people with a disability, from grass-roots to elite level; (3) attitudinal changes in the perception of the position and the capabilities of persons with a disability, as well as in the self-esteem of the people with a disability; and (4) opportunities for people with a disability to become fully integrated in social living and to reach their full potential in aspects of life beyond sports [14]. Furthermore, the International Paralympic Committee set the goal of para-athletes to inspire others, so others feel empowered and excited to participate in sport, and to further equity by challenging stereotypes, and by transforming attitudes and breaking down social barriers and discrimination towards people with disabilities [15]. Triathlon is one sport that comes with certain narratives around the sport, as a whole, and its athletes [16,17]. Paratriathlon made its debut in the 2016 Paralympics in Rio, and as such, paratriathlon plays a role now on the highest level in how parasport impacts disabled people. 

Given the new responsibility that comes with the new status of being a Paralympic sport and given the influence of newspapers, the objective of our study was to investigate the extent and nature of the coverage of paratriathlon and paratriathletes in Canadian newspapers present in the ProQuest database Canadian Newsstream, whereby we interpret our findings through the lens of the four legacy goals of the International Paralympic Committee, and what the International Paralympic Committee sees as the role of para-athletes.

### 1.1. The Legacy of the Paralympics 

The Olympics and Paralympics are increasingly concerned about their long-lasting legacy effect. The “International Olympic Committee (IOC) now requires long-term usage plans for new facilities as part of the bid process and a preliminary statement regarding the broader social, cultural, economic, and sports legacies that will result from hosting the games” [18]. Since 2007, the International Paralympic Committee identified legacy and legacy planning as important aspects of the hosting experience [14], whereby the four components of legacy are, as follows: “(1) Accessible infrastructure in sport facilities and in the overall urban development; (2) Development of sport structures/organizations for people with a disability, from grass-roots to elite level; (3) Attitudinal changes in the perception of the position and the capabilities of persons with a disability as well as in the self-esteem of the people with a disability; and (4) Opportunities for people with a disability to become fully integrated in social living and to reach their full potential in aspects of life beyond sports” [14]. Various Paralympics outlined what one could call legacy plans. According to Darcy in his part of the article, the Sydney Paralympic Organising Committee (SPOC) had, as primary goals, “raising awareness of the Paralympics; augmenting funds; keeping the momentum going after the Olympic Games, and attracting spectators to the Paralympics” whereas secondary goals were “a lasting legacy of accessible infrastructure (venues, transport, and accommodation), a raised level of disability awareness and an improved position in society” [19]. The 2010 Winter Olympic and Paralympic Games “goal of the Legacies Now programs centered upon enhancing community life—working, commuting, traveling, and playing through pointed initiatives and funded community projects to improve the quality of life for people with disabilities” [18]. The London 2012 Candidate file suggests that the Paralympic Games can “build respect … for disabled people by changing society’s perceptions” [20]. A 2011 poll indicated that 71% of the wider British public saw the potential of London 2012 to have a positive impact on disabled people’s lives however it was reported in the same article that disabled people remained skeptical of the London 2012 games [21]. 

However, the question is whether a legacy was also achieved, and whether it was and is durable. The International Paralympic Committee expects the organizing committee to allocate money to measure “the impact of the Paralympic games, as part of an overall project of measuring games impact for the host city, region and country” [14], which seems to be sensible in an area that increasingly asks for evidence-based policies. Darcy highlighted that “Politicians and the media contended that the Paralympics resulted in improved disability awareness amongst the non-disabled public” but that “no research was conducted to test this hypothesis before, during or after the Paralympics” [19]. A 2011 report on the 2010 Winter Olympic and Paralympic Games [22] also gave no concrete numbers as to the impact of the 2010 Winter Olympic and Paralympic Games since 2010. Misener argued that the framing of Paralympic legacy in the media “centered upon ‘othering’ athletes with a disability through the supercrip narrative” and the legacy issues presented did “not represent a broadening of the scope of the legacy of the Paralympic Games and, in fact, the critical role of the media in reframing the discourse about disability and accessibility was largely absent from the media frames” [18]. Conflicting narratives exist around the London 2012 Legacy. According to a 2015 UK government report, London 2012 provided an opportunity to drive forward the cause of disability equality by changing attitudes, improving access, and opening up new possibilities across sport, culture, and business [23]. However, this narrative is contested by various polls performed in the United Kingdom on behalf of disability organizations [24,25,26] and various academic studies [27,28,29] raising questions about disability equality following the Paralympics. As to the vision of the legacy of Tokyo 2020, Ogura highlights that an opinion poll conducted by the Cabinet office in 2015 indicated that according to 44.4% of the citizens polled, one legacy of the Tokyo 2020 Paralympic games should be “the enhancement of social understanding of disabled persons” [30], followed by 38.4%, that envisioned an improvement in the accessibility and barrier-free facilities, and 32% mentioned effects on the economy and employment [30]. Ogura further highlighted that some of the resistance to hosting the Tokyo 2020 games was linked to the belief that the focus is too much on competitiveness and medals, and not enough on increasing access for the “ordinary citizen in sport activities” [30]. Ogura outlined the Paralympic games-related content of The Tokyo 2020 Action & Legacy Plan 2016 Interim report from the Tokyo organizing committee of the Olympic and Paralympic games, which emphasized the importance of an inclusive society through “(a) increase of social awareness and recognition of disability sport, (b) improvement in the sport environment for the disabled community, (c) enhancement of social awareness for the welfare of disabled persons, (d) improvement in physical accessibility in arenas, stations and other facilities by promoting universal design and (e) promotion of arts activities of disabled people by organising various cultural events including wheelchair fashion shows” [30].

### 1.2. Legacy of the Paralympics and the Media

As to legacy and the role of media, one study concluded that there was no lasting legacy in relation to a change in the bias towards reporting on men versus women’s sports in the British print media with the London 2012 Olympics and Paralympics [31]. A book highlights mixed results as to the positive legacy of the media in the London 2012 Paralympics [32]. Crow questioned the use of the advertising campaign “Meet the superhumans”, which became the London 2012 Paralympic mantra [28] see also [33] and the ambiguity of a long term positive legacy claim is highlighted by [34]. The mixed legacy effect of various Paralympics with a special focus on the London 2012 Paralympics and the role of the media is outlined in [35]. Misener, in her study, concluded that the media covering of the Vancouver 2010 Winter Paralympics paid little attention to legacy concepts and the framing centered “upon ‘othering’ athletes with a disability through the supercrip narrative” [18] and “the critical role of the media in reframing the discourse about disability and accessibility was largely absent from the media frames” [18].

### 1.3. The Role of Athletes

Social and civic responsibility is a concept increasingly applied to sport and its athletes [36,37,38,39,40,41,42], including parasport and its athletes [43,44,45,46]. Athletes have an integral role in helping organizations increase awareness and create positive associations for their products and services, all the while building their own personal brand, and athletes have the ability to influence consumers’ perceptions towards a product, service, or organization [47]. Athletes are seen as agents of change [48] and as active citizens [48]. Elite sports are regarded as one of the vehicles for attracting young children into sport because of the inspirational function of elite athletes as role models towards youth, and the effects of international sporting success on the increasing public interest in sport [49]. According to Grix and Carmichael, “Elite sport success is seen to lead to both international prestige for the nation, a ‘feel-good factor’ among the population and, importantly, to an increase in participation among the masses. This, in turn, leads to a healthier nation and to a wider pool of people from which to pick the champions of the future” [50]. According to the International Paralympic Committee, some of the social responsibilities (although they do not use the term) of para-athletes are to inspire others, so others feel empowered and excited to participate in sport, to further equity by challenging stereotypes, and by transforming attitudes and breaking down social barriers and discrimination towards people with disabilities [15]. 

### 1.4. Paratriathlon 

Paratriathlon consists of a sprint distance of 750 m swim, 20 km bike (handcycle/tandem) and 5 km run (wheelchair) [51]. Paratriathlon is one of the two disciplines added to the Paralympics in Rio 2016, which increases its public profile and its footprint at the Tokyo 2020 Paralympics will be bigger [52]. 

Given that paratriathlon is now part of the same narrative around legacy and social responsibility of parasport, and given the reported negative aspect of the media coverage of parasport and para-athletes, we searched two academic databases, namely EBSCO-host—an umbrella database that consists of over 70 other databases, including sport-related ones such as SPORTDiscus, and Scopus, which contains all Medline articles for the keywords “paratriathlon” “para-triathlon”, “paratriathlete” and “para-triathlete”, limiting our searches to the “all fields” (Scopus) and “all text” (EBSCO) search categories, to investigate whether a media analysis has been performed around paratriathlon. After elimination of duplicates within and between the academic databases, we only found 27 academic articles. 

As to media, no article engaged with the newspaper coverage of paratriathlon. One article investigating the visibility of Paralympic sports on twitter during the Rio Paralympics 2016 stated that paratriathlon was one of the most visible subjects on Twitter on the 11 September 2016 [53], but the article did not provide content as to what was covered in the tweets. One study interviewed parasport participants, including paratriathletes (no numbers were given, but Meyrick and Shelley Ann were identified as paratriathletes) “to provide an empirical account of how the affordances of the web are leveraged in disability sport networks. Our findings suggest that individuals with disabilities are using blogs, Facebook, Twitter, and other forms of online communication to find information, engage in advocacy and outreach projects, and form strong networks that extend online and offline” [54]. 

The remaining articles contained non-media-related content covering the Triathlon Australia Selection Policy [55], physiological aspects, such as effect of caffeine [56], ventilatory threshold [57], anaerobic threshold [58], physiological attributes and training practices of elite paratriathletes [59,60], adaptive sports ankle prosthetics [61], and nutritional supplement use of para-athletes, which included 23 paratriathletes [56]. One article investigated organizational stressors encountered by athletes with a disability, which involved 5 paratriathletes reporting on 316 organizational stressors [62]. “Incompatible coaching style, unfair selection processes, the structuring of events at competitions and expectations to get a medal” [62] were reported as stressors for athletes with and without a disability. Stressors, unique to the disabled athletes, that were highlighted were “inaccessible venues for disability requirements, lack of disability-specific coaching and training, captaincy role pressure to understand different disabilities, lack of crowd at disability events, distractions of Paralympic Games being first international competition, costs of disability specific equipment, transitions from able-bodied to disability sport and the disability classification system” [62]. One study interviewing 14 paratriathletes looked at the “relevance of key components of Organismic Valuing Theory of Growth through Adversity in understanding posttraumatic growth amongst paratriathletes with acquired disability” [63] whereby the authors reported that “initial response to disability was largely negative, paratriathlon experiences were reported to be a mechanism through which growth was facilitated. In particular, participants suggested that social, competence, empowerment, and identity development processes were instrumental in facilitating posttraumatic growth” [63] and that “fostering perceptions of competence, autonomy, and social connection may promote posttraumatic growth” [63]. Only three articles mentioned social aspects around sport and disability. Colette Conroy argued that the Paralympic athlete profile is, in essence, an impairment profile [64], giving as an example that the paratriathlete profile on the International Triathlon Union webpage [65] uses language such as “medically diagnosed condition that causes a permanent impairment that can be measured objectively” [65]. Conroy made the case that the Paralympics is only allowing people with certain impairments to compete [64]. Weedon covering the aspect of nationalism and sport gave voice to the paratriathlete Stockwell stating “Stockwell reflected on her successes as a swimmer in the 2008 Beijing Paralympics and world champion paratriathlete in the context of her life-long patriotism” [66]. 

## 2. Method

### 2.1. Study Design

Framing is one way to perform a newspaper analysis [67,68]. We were interested what frames were used to describe paratriathlon and paratriathletes. Structural [69], content [70], and issue-specific framing [71,72] are three ways of framing. We were interested in how paratriathlon and paratriathletes are engaged with in the content of the newspapers. Our content analysis focuses on how the communicator (the newspaper) frames paratriathlon and paratriathletes. Persuasion is one media effect, which encompasses the message, who is used as a source and the “persuadability of media consumers” [73]. The question is what the reader will be persuaded of after reading the coverage of paratriathlon and paratriathletes in the newspaper articles? Misener, performing a framing analysis of how Canadian media framed the legacy of hosting the 2010 Winter Paralympic Games in Canada, noted that “media play an important role in shaping representations of disability and empowering social change” [18]. The question is what the take home message will be for the reader in relation to disabled people and social change? We interpret the data we obtained through the lens of the four legacy goals of the International Paralympic Committee, and what the International Paralympic Committee sees as the role of para-athletes.

### 2.2. Data Sources and Data Collection

To answer the research question, we used the *Canadian Newsstream*, a database consisting of 300 English language Canadian newspapers, which includes the two Canadian newspapers with national reach, *The Globe and Mail* and the *National Post*, and wire feeds (Canadian news wire and Canadian Press) as sources, for its complete time range from 1980 to December 2017. Articles from the *Canadian Newsstream* database were collected using the University of Calgary’s ProQuest online database. We accessed the ProQuest *Canadian Newsstream* database on December 1st 2017, limiting our searches to the presence of the key words “paratriathlon”, “para-triathlon”, “paratriathlete” or “para-triathlete” in the full text of newspaper articles present in the *Canadian Newsstream*. The obtained 167 articles (127 newspaper articles and 40 wire feed articles) were downloaded as one pdf file, and imported into ATLASti–8^®^ (Scientific Software Development GmbH, Berlin, Germany), a qualitative data analysis software application. We did not set any other inclusion or exclusion criteria, besides that the articles had to contain at least one of the keywords in the full text.

### 2.3. Data Analysis 

We provide some descriptive quantitative data on the publication timeline of the *n* = 167 articles, and which newspapers published how many articles. We employed a qualitative thematic content analysis of the 167 articles collected to answer the research question. There are many themes in a given newspaper article, whereby the themes do not have to relate to the subject investigated. The qualitative analysis was deductive in the sense that the themes had to relate to paratriathlon or paratriathletes. However, what themes emerged was not pre-set and, as such, could be seen as an inductive approach [74,75]. We developed a list of codes (covering the research question) in an iterative fashion, as we read the articles and themes based on the codes. We also provide numbers as to how often a certain qualitative theme was present. No statistics were employed. 

### 2.4. Rigor

Credibility/dependability and confirmability are three trustworthiness measures [76,77,78]. Both authors performed the initial search to obtain the target articles for qualitative analysis. Both authors obtained the same hit counts. The descriptive quantitative data presented is data obtained from the keyword search of the newspaper databases at the time the articles were selected. To enhance credibility/dependability, the authors engaged in peer debriefing on the generation of the qualitative data. Differences in codes and theme suggestions of the qualitative data were few, and discussed between authors and revised as needed. Confirmability is evident in the audit trail made by using memos and coding functions within ATLASti–8^®^. Regarding transferability, our method description provides all the necessary information for others to use if they decide to apply our keyword search to other English language media sources, or translate our keywords into other languages to search non-English sources. 

### 2.5. Limitations

The database searches were limited to the English language Canadian newspapers present in the *Canadian Newsstream* database. Therefore our results can not be generalized towards Canadian newspapers in general.

## 3. Results

### 3.1. Descriptive Quantitative Results

In the first step of our analysis we recorded the yearly hitcounts of articles we found for our search terms (Table 1).

Of the 167 articles obtained from the Canadian Newsstream that contained one of the terms: “para-triathlon”, “paratriathlon”, “paratriathlete”, or “para-triathlete”, 6 articles were reprinted in two newspapers, three were reprinted in four newspapers, and one was reprinted in five newspapers. Of the 167 articles, 127 of them were from a newspaper, and 40 from wire feeds. As to newspapers, the Edmonton Journal had 23 articles; Calgary Herald 12; Ottawa Citizen 10; The Province 6; and Star Phoenix 5. Forty newspapers had less than five articles each. As to the two newspapers with national reach, both the National Post and The Globe and Mail had two articles each. 

### 3.2. Qualitative Data

#### 3.2.1. Articles without Further Content

40 articles had no relevant content. Many articles did not provide content beyond mentioning that paratriathlon is or will be part of the 2016 Rio Paralympics; 23 articles mentioned triathlon/paratriathlon events without further content. 

#### 3.2.2. Major Themes 

The following were major themes, whereby we labeled a theme as major if present in more than 10% of the articles. 

##### Major Theme 1: Personal Story

Personal stories of paratriathletes, such as John Young, Leona Emberson, and Stefan Daniel, was the number one theme; for example, 50 of the 167 articles mentioned the Canadian triathlete “Stefan Daniel”. 

##### Major Theme 2: Inspire

48 articles contained 104 times terms with the root “inspir*”, covering words such as “inspiring”, “inspire”, “inspires”, and “inspiration”. To be inspired was, for example, linked to people doing more, and engaging themselves in recreational activities [79,80], and that one’s achievement are seen as inspiring [81].

To give one quote:

“We’re more about being out there and visible, giving people something to think about,” Cowie said, adding that words like, “inspiring, awesome” and “you guys rock” often come up in the conversation. “It’s humbling and overwhelming that people would stop and tell you what a difference you are making. You don’t expect it,” he said. However, on a deeper level, Cowie and Jones understood how their actions inspire and influence those around them. “It’s beginning to make a difference,” Cowie said, adding the reason the two strive to give back goes to the core of what is fundamentally good in life. “It’s like that hug you got when you were a little kid, and how it made you feel better,” he said. “It’s corny, but it’s really what the bottom line is. We do inspire people, we do motivate people, and if we do this two or three times, it gets people to think what they’ve done is a success.” Getting an email from a someone in reply to how he had helped him through a rough time is the motivation that keeps him going, Cowie said”.

[81]

##### Major Theme 3: Supercrip 

The supercrip theme highlights the achievement of person against large odds, and was the second biggest theme [81,82,83,84,85,86,87,88,89,90,91,92,93,94,95,96,97,98,99,100,101,102,103,104], whereby the supercrip theme was often linked to the personal story of the paratriathlete. The supercrip imagery was evident in wordings that one can do everything, for example “My motto is not can I? But how can I? [100], “can be just as fast as everyone else” [104], “no limits on what the human spirit can will you to do” [90]. The overcome imagery is another part of the supercrip imagery, and is evident in narratives such as “You have to take it for what it is and deal with it, and try to be happy with it” [102], “never thought of myself as disabled, or as a paraathlete” [82]; “Getting back in the saddle was never a problem” [92]; “it’s possible to accomplish tasks in spite of obstacles” [84]; “A little adversity or disability shouldn’t stop you” [84]; and “conquering everything despite all odd”[93]. Another example is: “You think I can’t? I’m going to prove you wrong. I’m going to figure out a way to do it” [99], whereby the athlete states that “It’s not about overcoming the disability, it’s about using every opportunity I have, taking it and going with it” [99]. 

To give one more quote:

“Mom, I’m a paraplegic.” Those were the first words Julian Nahachewsky uttered to his mother when she entered the hospital room after his accident. Before resting her head on his chest to pray, she calmly said: “Son, well, all the deep resources that you have within you—like strength and courage—that have been latent inside you, they are going to have to come alive now.”

And they did. Four years later, Julian is a high-level athlete. Just five months after his injury, he participated in the Canada Games. He’s competed across North America and Europe. He keeps beating his personal bests. He’s the first paraplegic to complete a triathlon in the province and he’s ranked 15th in the world for Para-triathlons. And he did it all without using his legs” [86].

##### Major Theme 4: Motivation 

Motivation was present as follows: “motivated to try and maximize his abilities”[86], motivated by the supercrip behavior of a relative, ““his older brother’s attitude is a source of motivation.” ”He’s been a pretty big inspiration of mine,” Daniel said from Victoria. “He never let his disability get to him”” [105], motivating others a goal, [81,88,106], taking part in able-bodied and parathletic disciplines a source of motivation [107]; winning able-bodied events a motivation [82], to cross the finish line [84], to walk again [84], athletes with challenges [108], success [108], family, [108], compete in junior elite triathlon [82], train with peers [109], competitors are motivated due to Rio approaching [110], and facing a challenge as the motivation [100]. 

##### Major Theme 5: Ability

Ability was another major theme whereby the term “able-bodied” was present in 24 articles. Often para- and able-bodies were mentioned in juxtaposition “para- and able-bodied athletes” [111], “as much power as an able-bodied swimmer” [112]. In the same way that there was the theme that one does not see oneself as disabled, there was the theme that one does not see oneself as a para-athlete “I’ve been training with able-bodied athletes my whole life, so I don’t really see myself as a para-athlete in training, anyways. That’s been a key for me in having success in able-bodied (events)—just not seeing myself as a para-athlete before races” [107]. Another ability theme was that the para-athlete wants to compete, is competing, against the able athlete: “Daniel would love to make the able-bodied team for the Olympics in Tokyo” [113]; “I’ve been able to compare myself every day to able-bodied athletes” [102]; “So now that Daniel is competing with able-bodied elite triathletes” [114]; and “‘I do love able-bodied racing,’ said Daniel. “I do want to compete at a high level in that, too. It is a goal of mine, for sure” [112]. Succeeding in able-bodied events is seen as particularly rewarding; “Daniel admits to being especially stoked after capturing Canada’s (able-bodied) junior elite triathlon championship” [112]; see also [107,115]. Part of the ability theme was that one wonders how one would have performed as an able-bodied athlete [107].

##### Major Theme 6 

Fifteen articles mentioned the performance in Rio.

#### 3.2.3. Minor Themes

##### Minor Theme 1: About the Canadian Paralympic Committee

Nine articles mentioned the Canadian Paralympic Committee *n* = 17 times.

“The Canadian Paralympic Committee is a non-profit, private organization with 46 member sports organizations dedicated to strengthening the Paralympic Movement. The Canadian Paralympic Committee’s vision is to be the world’s leading Paralympic nation. Its mission is to lead the development of a sustainable Paralympic sport system in Canada to enable athletes to reach the podium at the Paralympic Games. By supporting Canadian Paralympic athletes and promoting their success, the Canadian Paralympic Committee inspires all Canadians with a disability to get involved in sport through programs delivered by its member organizations. For more information, visit www.paralympic.ca”.

(for example, in [111,116])

##### Minor Theme 2: Access Issues

Access issues were mentioned in five articles as follows: access to paratriathlon training and programs, as well as partnership opportunities with the university [117], see also [118]; goal of the ParaSport Games is to “raise awareness of accessibility issues, lower barriers, and encourage more young people with a disability to leave the sidelines and participate in sport and recreation [119]; stating that there are many facilities in the county [80] and “Legacy facilities and equipment from the Toronto Pan and Parapan Am Games have also helped Canadian Paralympic athletes up their game”[120]. 

##### Minor Theme 3: Trickle-Down Issue

Trickle down as an issue was evident in that it was noted that there was the need to attract more Canadians to the sport [111,119,121]; to have more Canadians be involved in recreational activities [80,119,121] and “to become connected to the community” [121]. 

As to minor themes present in two articles, we found the demonization of wheelchairs, evident by the use of the term confinement [88], and indicating the importance of walking and that one does not want to be in the wheelchair [84]; a perception problem for the Paralympics (perception that it takes less effort to be part of the Paralympic team than the Olympic team [122], and the need for celebrating difference, [104]); the athlete wanted to die when the change in abilities happened [88,98]; cost issues [81,98]; and need for Triathlon recruitment [92,123]. 

Only one article described the paratriathlon: “Para-triathlon is practiced in 37 different countries: Athletes race in three disciplines: 750 m of swimming, followed by 20 km of cycling and 5 km of running. Competition categories are based on specific physical impairments. Athletes may use a hand cycle, tandem bicycle or bicycle in the cycling portion, and wheelchairs are permitted on the running portion of the course. Para-triathlon is unique in that it fully integrates athletes with physical and visual impairments alongside their able-bodied counterparts at the same event” [121].

## 4. Discussion

Our findings suggest that the paratriathlon and paratriathlete coverage in the Canadian newspapers covered employed mostly the overcoming personal challenges—the supercrip—theme using predominantly personal stories to anchor this theme. Another main theme was to compare para-athletes with able-bodied athletes. Some articles simply reported the results of events. Very few articles mentioned problems related to sport. No article linked paratriathlon and its athletes to broader societal aspects of sport, or mentioned the legacy goals of the Paralympics as an issue. The lack of mentioning of the legacy goals was also noted by a study that looked at the Canadian media coverage of the 2010 Winter Paralympics that took place in Vancouver, Canada [18]. The problematic coverage of Paratriathlon and its athletes, we found, is also in sync with the reporting on other para-athletes [12,13,33,124,125]. Our findings pose various problems for Paralympic sport and athletes, disabled sport on all levels, and disabled people in general. It hinders, for example, fulfilling the four components of the Paralympic legacy put forward by the International Paralympic Committee (IPC): “(1) Accessible infrastructure in sport facilities and in the overall urban development; (2) Development of sport structures/organizations for people with a disability, from grass-roots to elite level; (3) Attitudinal changes in the perception of the position and the capabilities of persons with a disability as well as in the self-esteem of the people with a disability; and (4) Opportunities for people with a disability to become fully integrated in social living and to reach their full potential in aspects of life beyond sports” [14]. Given that according to the International Paralympic Committee, some of the social responsibilities of para-athletes are to inspire others so others feel empowered and excited to participate in sport, to further equity by challenging stereotypes, and by transforming attitudes and breaking down social barriers and discrimination towards people with disabilities [15], with other words, that para-athletes are expected to be agents of change, our findings suggest that Paratriathlon athletes and their organizations have to come up with ways that decrease the reported problematic findings, in order to fulfill the legacy requirement of the Paralympics, and what the IPC expects from athletes. The remainder of this section discusses our findings through the lens of the four legacy goals, keeping in mind the social responsibility of para-athletes expected by the IPC, whereby we first discuss legacy goals 1 and 2 together, as they both focus on organizational issues, and then we discuss goal 3 and 4 together, as both focus on societal aspects. We finish the section by discussing what needs to be done by athletes and organizations.

### 4.1. Legacy Goal 1 and 2

(1) Accessible infrastructure in sport facilities and in the overall urban development; (2) Development of sport structures/organizations for people with a disability, from grass-roots to elite level.

#### 4.1.1. Legacy Goal 1 

Content mentioned in the newspapers that could further legacy goal 1 was, for example, the report that there is a need to give aspiring athletes access to paratriathlon training and programs [117]; see also [118]. One article made the point that “Legacy facilities and equipment from the Toronto Pan and Parapan Am Games have also helped Canadian Paralympic athletes up their game”[120], suggesting that the Toronto Pan and Parapan Am games have fulfilled the first part of legacy goal 1 of the Paralympics. At the same time, cost, which is one access issue, was only mentioned twice in the articles covered [81,98]. Furthermore, various articles reported in ways that do not further legacy goal 1; for example, one article mentioned that the goal of the ParaSport Games is to “raise awareness of accessibility issues, lower barriers, and encourage more young people with a disability to leave the sidelines and participate in sport and recreation” [119]. However, the article did not engage with how many disabled people do not participate in sport and recreation and why, and how the goal of the ParaSport Games could be achieved As written the reporting did not further the goal of the Legacy goal 1 because it (a) did not report on access issues, leaving the reader uninformed and unaware of the complexity of the problem, and (b) reported in ways that left the impression that just being a spectator at the ParaSport Games is enough to get involved in sport. Another article also covering the Parasport games argued that the action of the athletes will inspire people to get involved in recreational activities claiming, at the same time, that there are many facilities in the county [80], leaving the reader with the impression that there is no accessibility problem with the physically infrastructure or other issues, and that it is purely an issue of self-motivation of the disabled person. We could not find any article that thematized the accessibility issues of the urban environment which are part of legacy goal 1. Even worse, our finding that the wheelchair is demonized [84,88] which is in sync with other sport write-ups [12], could be seen as a negative for achieving legacy goal 1. 

#### 4.1.2. Legacy Goal 2 

As to legacy goal 2, not one article had content that related to the development of sport structures/organizations beyond that various articles suggested that paratriathlon will get a boost from now, being a Paralympic sport, and that including paratriathlon in the Paralympics will play “a significant role in attracting more Canadians to the sport regardless of age or disability” [111], without saying how that would make a difference. Furthermore, the reader is not made aware in any article that disabled people are underrepresented in sports on various levels, including the organizational structure [126,127,128]. From the articles, the reader will not have become literate on how parasport can best make a positive difference in the lives of people, a topic discussed within sport [48], or what the role of sport structures/organizations are. 

To sum up: the newspapers covered had no real content that would give the reader an idea that there are problems related to legacy goal 1 and 2, and that changes are needed. 

### 4.2. Legacy Goals 3 and 4

(3) Attitudinal changes in the perception of the position and the capabilities of persons with a disability, as well as in the self-esteem of the people with a disability, and (4) Opportunities for people with a disability to become fully integrated in social living and to reach their full potential in aspects of life beyond sports [14]. 

Our findings suggest that goal 3 and 4 are not only not advanced through the newspaper coverage, but actually hindered in two main ways. One negative impact on legacy goal 3 and 4 is linked to the excessive use of the supercrip theme [81,83,84,85,86,87,88,89,90,91,92,93,94,95,96,97,98,99,100,101,102,103,104], using personal stories to anchor this theme. Another negative impact is linked to the use of “able-bodied” as a term to describe non-para-athletes, which was present in 24 articles, whereby within these 24 articles, a main theme was that the para-athlete wants to compete against the able athlete [102,107,112,114,115], including Olympic athletes [112,113], and that one does not see oneself as a para-athlete [107]. 

#### 4.2.1. Supercrip 

As to the presence of the supercrip theme in the articles we investigated [81,83,84,85,86,87,88,89,90,91,92,93,94,95,96,97,98,99,100,101,102,103,104] and the many feel good personal stories, the findings are not surprising. The supercrip theme has been described and critiqued in relation to para-athletes by many [11,12,125,129,130,131]. Crow noted, for example, that the Channel Four mission as official broadcaster, and in keeping with government Paralympic legacy of the London 2012 Paralympics, was to “transform the perception of disabled people in society [28] and that Channel Four thought that the mandate was best served by using the slogan “Meet the superhumans” in the advertising campaigns which, indeed, became a Paralympic mantra [28], whereby Crow concluded that through this slogan, a “hierarchy of impairment was re-enacted. Foremost are amputees with high technology prostheses. For spectators, the transformative powers of technology mark the apotheosis of superhuman” [28]. Legg highlighted various problems with how we talk about Paralympic athletes, such as that the focus is more about the ability of the athlete to overcome obstacles then the sport-related achievement [132], and the reduction of Paralympic athlete achievement to feel-good stories [132]. 

#### 4.2.2. Able-Bodied

The term “able-bodied” was used over 24 times, in juxtaposition to the para-athlete, indicating the para-athlete to be the lesser-abled counterpart. If one group is able-bodied, then the other group has to be the non-able-bodied. Indeed, some academic and grey literature questions the use of the term able-bodied, “We refer to ‘non-disabled people’ rather than ‘able-bodied people’ as the latter term can have pejorative implications for disabled people” [133]. 

Themes that one does not see oneself as disabled [82], a theme that is also used by other para-athletes [134], and that one does not see oneself as a paratriathlete [107], we submit, are linked to the less-abled sentiment. One wonders whether the athletes using such statements understand the consequences of such statements. We submit that such statements set the stage for a negative connotation of disabled people, and para-athletes negating efforts related to legacy goals 3 and 4. How does it increase the self-esteem of a para-athlete if the narrative is of a less-abled-one? Indeed, the less-abled narrative has been critiqued to be also evident in the classification system of the Paralympics, and how one is eligible for the Paralympics [135]. According to the IPC, “the word “Paralympic” derives from the Greek preposition “para” (beside or alongside) and the word “Olympic”. Its meaning is that Paralympics are the parallel games to the Olympics, and illustrates how the two movements exist side-by-side” [136], which is not suggested by the ability juxtaposition, and not feeling like a para-athlete or negating one’s status as a disabled person. 

Another ability theme was that the para-athlete wants to compete, is competing, against the able athlete [102,107,112,113,115]. One wonders what the reaction would be if, for example, female athletes would see it as better to compete against male athletes than competing against other female athletes. One article quotes Lindsey Vonn wanting to compete against male skiers, however, this is not seen as a serious exercise, but a one-time gimmick whereby Vonn is quoted in the same article that she believes she would only be “averagely competitive” [137]. The story of Vonn seems to be different to the narrative of para-athletes wanting to compete against “able-bodied” athletes. 

### 4.3. What to Do? 

Media, including newspapers, influence discourses [138,139,140,141]. Some note that the role of media has increased in recent times [140]. Our findings suggest that the newspapers we covered influence discourses around parasport and sport for disabled people in a negative way, if we look at the newspaper coverage of paratriathlon through the eyes of the four legacy goals. So what should or could be done?

#### 4.3.1. Role of Canadian Paratriathlon and Triathlon Organization 

Looking at the strategic plan 2017–2020 of Triathlon Canada [142] through the lens of the four legacy goals raises a few questions, and suggests that certain actions should be taken based on our findings. 

The strategic plan has as a goal to “develop and deliver a comprehensive communications plan for 2017–2020 which identifies key stakeholders, ensures consistent messaging, builds trust and enhances accessibility” [142]. What is meant by enhancing accessibility? Is this purely about obtaining more paratriathletes or is the goal broader, with a societal mandate in sync with achieving legacy goal 1? What does consistent messaging mean? What messages are to be conveyed? How much do the messages encompass legacy goals 1–4? 

It is stated that “Triathlon Canada fosters a culture of excellence and achieves gold medal performances in every event and every endeavour” [142]. If a gold medal is everything, does this leave space for a message that would increase the success for legacy goal 2? 

Another goal is to “complete a communications audit of internal and external stakeholders to understand existing perceptions and identify gaps” [142]. What is meant by perception? Is it in sync with legacy goal 3?

Another goal is to “leverage current national events to enhance the sport’s reputation and build corporate awareness and partnerships” [142]. What is the scope of the vision of sport’s reputation? Is it about the high-performance success of paratriathlon, or is part of the sport’s reputation its influence on society at large as it relates to disabled people in sync with legacy goal 4? 

Another goal is to “build Triathlon Canada’s reputation as a leader amongst NSOs” [142]. Does this mean that Triathlon Canada, including its Paratriathlon aspect, has to move beyond focusing on itself but has to actively pursue legacy goals 1–4 which are societal focused?

Reading the strategic plan reveals that no wording is present to suggest an alignment of Triathlon Canada beyond its own sport and how to enable the four legacy goals of the Paralympics. The question is, if individual Paralympic sports do not write the Paralympic legacies into their goals, how successful will Paralympic sport be in fulfilling the legacy goals on a country level?

Our findings suggest that Triathlon Canada has to fine-tune its communication strategy in relation to its para-section, and train paratriathletes and other athletes involved in paratriathlon to understand what the consequences of certain statements are. Triathlon Canada also has to find ways to fulfill more actions linked to the four legacy goals. Although the IPC legacy goals are for cities that host the Paralympics, it seems to be logical that the para-related sport organizations work at least on local, regional, and national levels, to further the same goals. 

We could not find anything about training paratriathletes in how to deal with the media in the strategic plan. Given the findings of our study this might be a worthwhile addition to the strategic plan. Triathlon Canada has a social media policy [143]. But the content does not cover issues flagged as problematic in our study

To move beyond Canada, the strategic plan 2018–2021 of the International Triathlon Union [144] states “Develop a legacy in the Youth Olympic, Olympic & Paralympic cities”, but no content can be found in the strategic plan that could be seen to tackle the four legacy goals of the Paralympics. Furthermore, using the term cities in the quote suggests a limited approach. Indeed, if legacy goals are interpreted to be limited to the hosting city, this suggest the need to clarify that the legacy goals are not just hosting city-limited but also have societal aspirations. 

#### 4.3.2. Role of Athletes

In the report “Sporting Success, Role Models and Participation: A Policy Related Review” [145], it is stated:

A “sporting hero” is a person whom one admires. The hero is defined by a visible personification of certain traits. For example, they may be interpreted by the observer (and/or portrayed by the media) to demonstrate perseverance, to be self-effacing and modest, with social responsibility. The hero may have succeeded “against the odds”. Most of the time, the hero will display mastery in her/his sport, but can achieve at a range of local and national levels.

[145]

Our findings indicate that the theme of perseverance and succeeding against the odds were present in many articles [81,83,84,85,86,87,88,89,90,91,92,93,94,95,96,97,98,99,100,101,102,103,104]. Social responsibility as a theme was not evident.

According to the International Paralympic Committee, some of the responsibilities of para-athletes are to inspire others, so others feel empowered and excited to participate in sport, and to further equity by challenging stereotypes and by transforming attitudes and breaking down social barriers and discrimination towards people with disabilities [15], responsibilities that could be seen as social responsibilities. In the way the athletes are quoted in the newspaper articles, we do not think that the coverage made the athletes agents of change in regard to all the responsibilities. Although it is mentioned sometimes that an athlete inspired someone else to get involved in sport, we submit that most of the paratriathlon coverage and most quotes attributed to paratriathletes in the newspapers are not useful to challenge the problematic stereotypes, of people with disabilities and athletes with disabilities, in existence, or to transform attitudes or to diminish social barriers and discrimination towards people with disabilities. That it is stated that although the Paralympic visibility has increased, that the benefits have not trickled down to the average disabled person [9] should make one think. The Paralympics “should be seen as one window to celebrate difference and to marvel at the performances of the athletes that are out there. Not better or worse, but different” [104]. However, by allowing the juxtaposition with the term able-bodied, and the supercrip imagery, one does not engender the celebration of difference. We submit that many paratriathletes, and for that matter, para-athletes, might not be aware of the negative consequences of certain wording, such as when one says that one is not disabled. In addition, we do not think that the consequences of how a para-athlete talks about wanting to take part in non-para-athlete events is well understood. We suggest that paratriathletes might need training in how to appear in public, and how one avoids getting instrumentalized. Furthermore, if legacy goals 1–4 are to be fulfilled, we need more discussions on how much athletes including paratriathletes have thematized and should thematize the societal problems, as evident in legacy goals 1–4; what barriers they face if they want to mention societal problems, as evident in legacy goals 1–4; what their role is and should be as agents of change; and what their social and civic responsibilities should be. We submit that we have to understand better the role of para-athletes including paratriathletes in society, and in regard to societal issues faced by people with disabilities. We need better best practices for being agents of change. More work has to be done to have workable best practice that decreases the problematic coverage in, for example, the newspapers, as it seems existing best practices fall somewhat short, at least in the newspaper articles we covered.

## 5. Conclusion 

Given that paratriathlon is now a Paralympic sport, the interest in reporting on paratriathlon, and the impact of such reporting on disabled people, increases. We submit that the coverage of paratriathlon we found has a negative impact on the fulfillment of the four legacy goals. Furthermore, the reader will not have become literate on any problems indicated by the four legacy goals [14]. A 2010 Canadian Press article stated:

“As an athlete and sports administrator, Elisabeth Walker-Young is thrilled by the increase in participation at the Paralympics and the awareness the Games generate for people with a disability. What still frustrates her is how this growth at the elite level has not “trickled down” into more people with disabilities becoming active. Statistics Canada estimates only three per cent of people who say they have a physical disability consider themselves physically active. Some disabled people may feel uncomfortable at a gym or pool because of the looks they receive”.

[10]

The quote highlights the failure of legacy goals, so far, and the need for actions.

Although tools are developed to aid managing athletes’ levels of personal and social responsibility [40], our findings suggest that it might be warranted to train paratriathletes on what to say in the media, in order to be aware of the danger of one’s statements being misused, for example, by being put into a context not wanted by the paratriathlete. Paratriathlon organizations with their athletes have to think even more about their media strategies. Having the media present the sporting achievements in a factual way, and eliminating the use, by media, of personal stories, could be one aspect of the strategy, but it is not enough if legacy goals 1–4 are to be fulfilled. If legacy goals 1–4 are to be fulfilled, we need more discussions on how much athletes or their organizations have thematized the societal problems reflected in the focus of legacy goals 1–4, and to provide strategies for fixing the problems. We need more data on whether social issues were raised by paratriathletes and para-athletes, in general, but were simply not reported by the newspapers. Social responsibility of athletes [37,38,39,40,41,146,147] and the role of athletes as activists [148,149,150,151] and agents of change [6,48] are increasing areas of research, which we suggest might be useful to expand toward paratriathlon and its athletes. Furthermore, it is often said that newspapers are not needed anymore in the time of social media. Although such a claim can be contested, it might be useful to look into whether Facebook pages or Twitter accounts, for example, related to paratriathlon or other sport actively thematize the legacy goals in a consistent ongoing fashion. It is well known that education is an important part, in order for sport to achieve its full positive potential [48]. It might be useful to ascertain what the impact of the paratriathlon coverage in the newspapers is on the views of youth on disabled people, disabled athletes, and the role of sport and its athletes in the world and in relation to disabled people. 

## Figures and Tables

**Table 1:** Search strategy and timeline of articles found.

Search Terms	Pub Date 1980–2008	2009	2010	2011	2012	2013	2014	2015	2016	2017	Total
“para-triathlon” OR “paratriathlon” OR “paratriathlete” OR “para-triathlete”	0	1	10	5	12	31	21	20	61	7	167
